# Commentary: Bridging the silos: A comparative analysis of Implementation Science and Improvement Science

**DOI:** 10.3389/frhs.2022.964489

**Published:** 2022-09-21

**Authors:** Nicoline Vackerberg, Ann-Christine Andersson

**Affiliations:** ^1^Jönköping Academy for Improvement of Health and Welfare, School of Health and Welfare, Jönköping University, Jönköping, Sweden; ^2^Qulturum, Center for Learning and Innovation in Healthcare, Jönköping, Sweden; ^3^Department of Care Science, Faculty of Health and Society, Malmö University, Malmö, Sweden

**Keywords:** Improvement Science, Implementation Science, quality improvement, pragmatism, positivism

The commented paper offers a comparative analysis of Implementation Science and Improvement Science, aiming to identify if these fields could benefit from and enrich each other. The method used is a systematic literature review.

In this article, the authors recognize that both fields have different origins and draw on different bodies of knowledge, however they claim that both Implementation Science and Improvement Science can be positioned within a positivistic tradition with some interpretive features. We challenge this claim, as positivism has ties to natural science and works within a very standardized context (1, 2), an objective methodology seeking to discern what is true or false. Implementation Science can probably be defined as partly positivistic, aiming at introducing evidence-based practice, which in turn is dependent on positivistic studies, verifying a hypothesis using experimental designs (2), mainly RCT. Implementation Science is trying to understand and explain the conditions for implementation, relying on existing theories or developing new ones from evidence-based facts. Improvement Science, on the other hand, examines whether quality improvement in health and welfare settings results in better clinical practice, patient, and population outcomes (3), with a focus on usefulness. Improvement Science attempts to develop and test program theories that can improve the understanding of assumptions and increasing awareness of what is done and why (4). Therefore, Improvement Science is more aligned to pragmatism, where the focus is not just on producing knowledge but on trying to adapt and transform knowledge in different contexts to make clinical practice better, to achieve usefulness. We argue that it is important to view the distinction between the somewhat more positivistic Implementation Science and the truly pragmatic Improvement Science due to methodological issues. Evidence of QI benefits is sometimes criticized for lack of rigor (3) and not being based in RCT methodology. To gain an understanding of what value Improvement Research contributes, it must be seen from a pragmatic lens.

We argue that Improvement Science therefore demonstrates a more a pragmatic than positivistic view, focusing on what works for whom, where, and when (5). One can also argue that there are elements of a social constructivist view in both Implementation and Improvement Research. Trying out better ways of working, new routines, and implementing them, are constructions by persons in the system, and require structured methods to evaluate the outcome. In both fields, context is influencing the result, which also challenges the positivistic positioning of the authors, since natural science tries to eliminate any circumstances that could bias the studies.

Another challenge to the authors' stance is that theories in Implementation and Improvement Sciences are developed from a range of research fields like psychology, nursing, organizational behavior, and sociology. These fields focus on objective facts while taking human factors, like change psychology, into account and relate much more to social constructivism and pragmatism than to positivism. There are similarities between positivism and pragmatism, but also large differences. An important difference, as we interpret it, is the origin of knowledge (ontology and epistemology). In the positivistic tradition, there is a strong belief in what is right or true (1, 2). This belief resonates more with Implementation Science, wanting to implement the already known most evidence-based way of doing things. Improvement Science, on the other hand, relies more on describing what works—for whom, where and when (5), even if that description is not taken to be the truth—at least not an overarching truth everywhere. This focus also implies that Improvement Research needs more interactive research approaches (6) to contribute to immediate usefulness in practice, which also corresponds more to a pragmatic view.

In light of the discussion above, we are wondering why the authors position themselves in the tradition of positivism. Could it be that there still is a believe that positivism is highly “ranked” in the medical world, where improvement and implementation will contribute? If so, we think that improvement and implementation researchers need to do even more research to show that the pragmatic usefulness is important when improving quality and patient safety in health and welfare (3).

We believe the commented paper adds some interesting comparisons and reflections about the connection between Implementation and Improvement Science, but think it is unhelpful to position them in the positivistic natural science tradition. Instead, these two research areas need to be understood in their complexity and their value tied to their ability to combine evidence-based facts with change management and behavioral theories. It requires an openness concerning contextual, cultural, and individual details and variation. Therefore, we argue that Implementation Science and especially Improvement Science are better positioned in the pragmatic tradition, where focus is not just to produce knowledge but trying to explain the usefulness of knowledge in different contexts in order to improve practice and outcomes.

## Author contributions

All authors listed have made a substantial, direct, and intellectual contribution to the work and approved it for publication.

## Conflict of interest

The authors declare that the research was conducted in the absence of any commercial or financial relationships that could be construed as a potential conflict of interest.

## Publisher's note

All claims expressed in this article are solely those of the authors and do not necessarily represent those of their affiliated organizations, or those of the publisher, the editors and the reviewers. Any product that may be evaluated in this article, or claim that may be made by its manufacturer, is not guaranteed or endorsed by the publisher.
